# Dose-volume parameter evaluation of a sub-fractionation workflow for adaptive radiotherapy of prostate cancer patients on a 1.5 T magnetic resonance imaging radiotherapy system

**DOI:** 10.1016/j.phro.2025.100706

**Published:** 2025-01-30

**Authors:** Georgios Tsekas, Cornel Zachiu, Gijsbert H. Bol, Jochem R.N. van der Voort van Zyp, Sandrine M.G. van de Pol, Johannes C.J. de Boer, Bas W. Raaymakers

**Affiliations:** Departement of Radiotherapy, University Medical Center Utrecht, Heidelberglaan 100, 3584 CX, Utrecht, The Netherlands

**Keywords:** Prostate cancer, MR-linac, Adaptive radiotherapy, Adapt-to-position, Adapt-to-shape, Intrafraction motion, Motion management, Hypofractionated radiotherapy

## Abstract

**Background and purpose:**

This study focuses on evaluating a sub-fractionation workflow for intrafraction motion mitigation of prostate cancer patients on a 1.5 T magnetic resonance imaging radiotherapy system.

**Materials and methods:**

The investigated workflow consisted of two sub-fractions where intrafraction drift correction steps were applied based on a daily reference plan. However, the daily contours were only rigidly shifted to match the intrafraction anatomies and therefore the clinical dosimetric constraints might be violated. In this work, daily contours were deformed to match the intrafraction anatomies and the online plans were re-calculated for a total of 15 patients. The deformed prostate contours were inspected by radiation oncologists and corrections were performed when necessary. Finally, a dose-volume parameter evaluation was performed on a sub-fraction level using the clinical plan parameters.

**Results:**

Clinically acceptable coverage was reported for the target structures resulting in mean V_95%_ of 99.7 % and 97.8 % for the clinical target volume (CTV) and planning target volume (PTV) respectively. Sub-fractions with insufficient CTV dose can be explained by the presence of intrafraction rotations and deformations that were not taken into account during intrafraction corrections. Additionally, for no sub-fraction the dose to the organs-at-risk exceeded the clinical constraints.

**Conclusion:**

Given our results on the CTV coverage we can conclude that the sub-fractionation workflow met the dosimetric constraints for the hypofractionated treatment of the analyzed group of prostate cancer patients. A future dose accumulation study can provide further insights into the suitability of the clinical margins.

## 1 Introduction

Magnetic resonance imaging (MRI)-guided radiotherapy offers high soft tissue contrast and thus promises precise and anatomy-tailored radiotherapy treatments. The clinical introduction of MRI linear accelerators (MR-linacs) [1], [2] has led to adaptive treatment workflows that allow for daily plan adaptations and online plan re-optimization to account for any anatomical changes while the patient lies on the treatment couch [3], [4].

The superior soft tissue visualization that the MR-linacs offer has also facilitated the move towards dose escalation and hypofractionated treatments [5]. Prostate cancer is one of the tumour sites that can greatly benefit from hypofractionated treatments [6], [7], [8], [9] that require less hospital visits for the patients [10] and can offer superior oncological outcomes [11], [12]. Yet, hypofractionation comes at the cost of longer treatment sessions, which can lead to larger treatment uncertainties due to intrafraction motion. Prostate intrafraction motion occurs predominantly in the superior-inferior and anterior-posterior direction due to bladder filling and gas pockets [13], [14], [15], [16]. Additionally, prostate and seminal vesicle rotations can occur at an intrafraction timescale [16]. Therefore, motion mitigation strategies have to be developed to counteract intrafraction motion events to achieve the high accuracy desired in these treatments [17], [18].

Previous work has focused on the development and clinical introduction of a sub-fractionated treatment workflow that delivers 7.25 Gy in two daily sub-fractions of 3.625 Gy each for a complete treatment scheme of 5 x 7.25 Gy [19]. The rationale behind this workflow was that splitting the fraction in two parts and performing additional online plan adaptions will help reduce the effects of intrafraction motion without the need for dose accumulation. In the clinical sub-fractionation workflow two rigid plan adaptation steps were performed after a daily full plan re-optimization step [3]. Rigid plan adaptations are an efficient technique to account for prostate drifts that occur during the treatment session by matching the daily pre-treatment MRI scan with an additional online position verification (PV) scan using translation-based registration. Nonetheless, the underlying anatomical changes cannot be purely explained by rigid 3D translations, as rotations and deformations will also be present. Additionally, the shifted contours were not deformed to match the PV anatomies during the clinical workflow, hence the online plan assessment might be inaccurate.

This work focused on assessing the accuracy of the clinically administered plans of the investigated sub-fractionation workflow using corrected contours that accurately match the online PV anatomies. Thus, the investigated workflow could be evaluated in terms of target and organs-at-risk (OAR) coverage and its treatment efficacy in the context of hypofractionated prostate radiotherapy could be assessed. We believe that the outcomes of this work are relevant for a variety of clinical workflows aiming at intrafraction motion management.

## 2 Materials and methods

### 2.1 Clinical workflow

The offline planning phase was performed using an MRI-only workflow: A pseudo CT was generated from a T2-weighted (T2w) MRI, using bulk electron density assignment, where the bones and femur heads were assigned the average value per contour and the rest of the patient anatomy was set to 1.0.

**Fig. 1:**
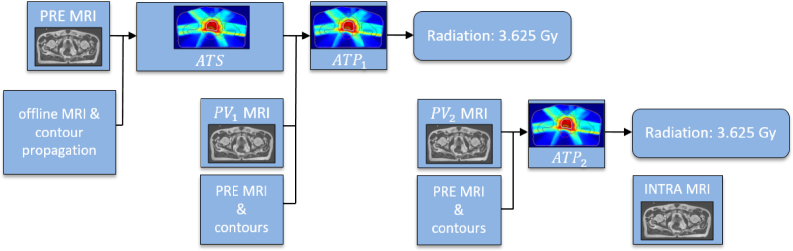
Overview of the investigated sub-fractionation workflow. Abbreviations: ATS: adapt-to-shape, ATP: adapt-to-position, PV: position verification.

The clinical sub-fractionation workflow investigated here (Fig. 1) [19] consisted of an initial daily online T2w MRI scan (PRE), which was registered using deformable image registration to the offline MRI and the offline contours were automatically propagated to the daily anatomy. If needed, the contours were manually adjusted by the operator (contours_PRE_). Then, an adapt-to-shape (ATS) step [3] was performed, where the offline plan was re-optimized based on the daily anatomy and the contours_PRE_. For the ATS step, all contours (including bones) were propagated using deformable image registration.

After the ATS step, a PV T2w MRI scan (PV_1_) was acquired and an adapt-to-position (ATP) step [3] (ATP_1_) was performed to account for rigid motion between the PRE and PV_1_ anatomy. The isocenter of the plan was then updated based on the results of a translation-based registration between the PV_1_ and the PRE MRI. The ATP step thus mimics a rigid translation of the initial ATS dose prior to re-optimization, similar to a virtual couch shift [20]. After the dosimetric coverage of the ATP_1_ plan was assessed and approved using the contours_PRE_, the first half of the prescribed radiation dose was delivered, while another PV scan (PV_2_, T2w) was acquired simultaneously. Then, a second ATP step (ATP_2_) was applied and was approved using the contours_PRE_ in a similar manner to the ATP_1_. Finally, the second half of the treatment was delivered and a final T2w MRI scan (INTRA) was acquired for the validation of intrafraction motion based on the measured displacement during the delivery.

### 2.2 Patient data

A total of 15 patients with intermediate-risk prostate cancer that underwent hypofractionated radiotherapy (5 x 7.25 Gy) with the daily sub-fractionation workflow in our clinic were included in this analysis. All patients completed their treatment between August and December 2023. A medical ethics approval was granted for acquiring, processing and publishing the patient data and results (MOMENTUM study, ClinicalTrials.gov ID: NCT04075305).

The beam configuration included seven beams at 0°, 50°, 100°, 155°, 205°, 260° and 310°. Each patient received 5 daily fractions, each consisting of 2 sub-fractions, thus resulting in 10 delivered plans per patient and a total of 150 plans. One patient fraction (2 sub-fractions) was excluded from our analysis, since the approved ATS contours for the femurs and pelvic bones appeared misaligned with the underlying patient anatomy, resulting in a total of 148 sub-fractions.

The average time between the PRE MRI and PV_1_ MRI was 15.2 min. The corresponding time interval between PV_1_ and PV_2_ MRI was 7 min and between PV_2_ and INTRA 8 min on average. Average beam-on time was 10.9 min per daily treatment (for the delivery of 2 sub-fractions). The average 3D displacement, as measured from the isocenter shift in the adapted plan, between PRE and PV_1_ anatomies was 3 mm ± 2.2 mm (5th percentile: 0 mm, 95th percentile: 6.7 mm, interquartile range: 2.6 mm), while the corresponding 3D drift between PRE and PV_2_ anatomies was 3.5 mm ± 2 mm (5th percentile: 0 mm, 95th: percentile 7 mm, interquartile range: 3.2 mm).

### 2.3 Research workflow

Since the contours on the PV scans are not manually corrected by the operator in a clinical workflow, they might not accurately match the underlying PV anatomies. Therefore, the clinically performed evaluation of the ATP plans might be inaccurate. Thus, in the scope of this work, a deformable image registration research algorithm was used to estimate the anatomical deformations and propagate the contours_PRE_ to the PV anatomies.

The deformation vector fields (DVF) between the PRE MRI and the PV_1_/PV_2_ MRI scans were calculated using an in-house developed deformable image registration algorithm [21]. The estimated DVFs were used to deform and propagate the contours_PRE_ to the corresponding PV anatomies, thus creating contours_PV_1__ and contours_PV_2__.

Three experienced clinicians evaluated the propagated prostate gland contours of all patients on PV_1_ and PV_2_ scans and corrections were performed if needed. The gross tumour volume (GTV) contours, which represent the DWI-dominant lesion, could not be assessed using only a T2w MRI scan, thus the automatically propagated contours were accepted.

After the contours_PV_1__ and contours_PV_2__ were checked, the corresponding dose distributions on the PV_1_ and PV_2_ anatomies were re-calculated using an in-house developed, GPU-based Monte Carlo dose engine [22]. For the rest of this manuscript the re-calculated dose files using contours_PV_1__ and contours_PV_2__ will be referred to as “corrected” cases. Similarly, the clinically approved ATP dose distributions will be referred to as “approved” cases and were also re-calculated, alongside with the ATS, corrected ATP1 and corrected ATP2 dose distributions using the same dose engine for ensuring consistency in voxelization. A statistical uncertainty of 3 % per control point was used in our dose calculations to match the clinically used settings.

Table 1 presents the differences in calculation of ATS, approved ATP and corrected ATP dose distributions: In the ATS case, contours_PRE_ and the ATS plan were used. The approved ATP dose distributions (approved ATP_1_, approved ATP_2_) reflect what was approved in clinic: dose distributions calculated using an ATP plan, evaluated on the daily contours_PRE_, without any contour correction. Finally, the corrected ATP dose distributions (corrected ATP_1_, corrected ATP_2_) were re-calculated using the ATP plans and contours_PV_1__, contours_PV_2__.

**Table 1:** An overview of the different cases used for re-calculating the dose distributions. Abbreviations: **contours_PRE_**: The daily contours of the PRE anatomy, **contours_PV_1__**: The corrected contours of the PV_1_ anatomy, **contours_PV_2__**: The corrected contours of the PV_2_ anatomy.

Case	Anatomy	Plan
ATS	contours_PRE_	ATS
approved ATP_1_	contours_PRE_	ATP_1_
corrected ATP_1_	contours_PV_1__	ATP_1_
approved ATP_2_	contours_PRE_	ATP_2_
corrected ATP_2_	contours_PV_2__	ATP_2_

### 2.4 Dose-volume analysis

For the dose-volume assessment, the corrected prostate contours were used to re-create the GTV+4mm, clinical target volume (CTV) and planning target volume (PTV) structures on the PV_1_ and PV_2_ anatomies using the clinical margin recipe: The GTV+4mm was created by expanding the GTV contour using an isotropic 4 mm expansion, while staying outside of the rectum and bladder contours. Then, the CTV was created by adding the GTV+4mm structure to the prostate contour. Finally, the PTV was created by expanding the CTV using the clinically applied margins: 2 mm in the left–right and superior-inferior directions and 3 mm in the anterior-posterior direction [19].

For the clinically relevant OARs (bladder, rectum and sphincter) we used the automatically propagated contours provided by the image registration algorithm for our analysis, after visually checking them for large mismatches. The V_95%_ (V_3444 cGy_) and dose-volume-histograms (DVH) were calculated for the re-created CTV and PTV target contours and propagated OAR contours. Table 2 presents the clinical constraints used in our department for intermediate-risk prostate cancer patients treated using the sub-fractionation workflow on a 1.5 T MRI radiotherapy system.

We investigated the dosimetric differences between corrected and approved ATP cases with focus on the planning goals (Table 2) for the target and OAR structures. ATS, approved ATP and corrected ATP cases were analyzed per sub-fraction. For our analysis, individual sub-fractions were assessed using the clinical constraints of the complete treatment scheme. In addition, we calculated the dosimetric differences for the D_5cc_ of the bladder and D_1cc_ of the rectum between approved and corrected ATP dose distributions per sub-fraction to investigate potential over-dosage in individual cases.

**Table 2:** Clinical constraints for the target and OARs. These constraints correspond to the full fractionation scheme of 5 × 7.25 Gy. The tolerance values in parenthesis represent the lowest values that would still be acceptable for clinically approving a plan of an individual ATP sub-fraction (98.9 % for the CTV and 96 % for the PTV). Clinically approved ATS plans typically achieve higher PTV coverage.

Organ	Constraint (with tolerance)
CTV	V_3444 cGy_ > 99 % (−0.1 %)
PTV	V_3444 cGy_ > 99 % (−3 %)
Bladder	D_5cc_ < 3700 cGy
Rectum	D_1cc_ < 3800 cGy
Sphincter	D_mean_ < 2000 cGy

## 3 Results

**Fig. 2:**
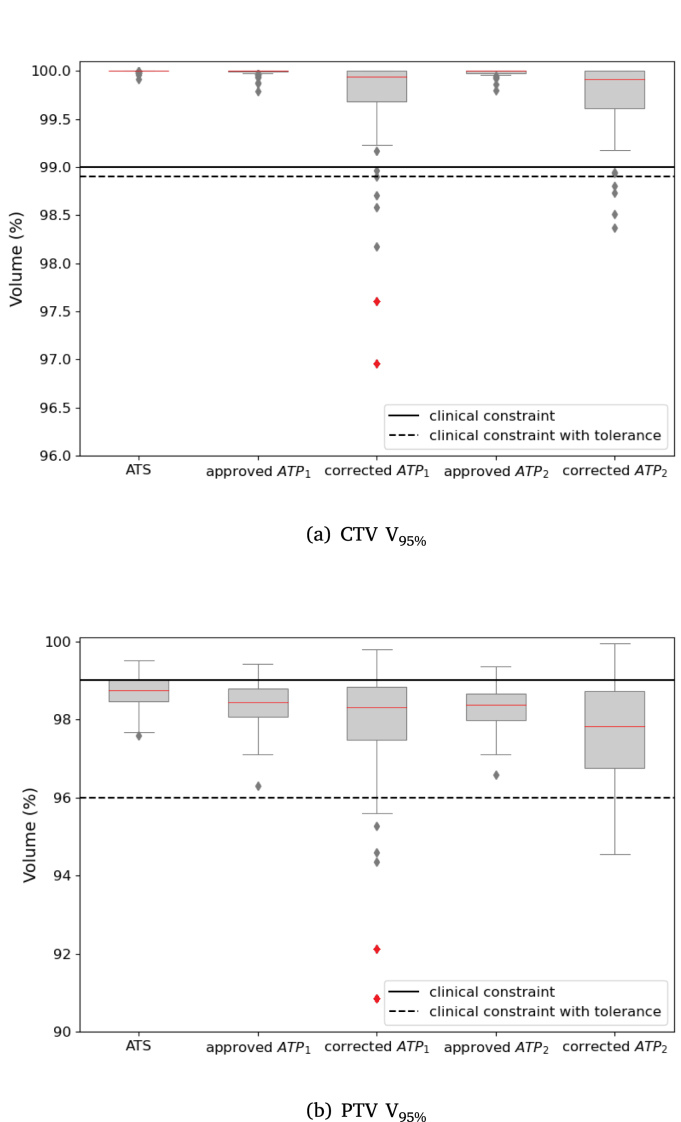
CTV and PTV coverage for the ATS, approved ATP and corrected ATP cases, evaluated on the corresponding anatomies (Table 1). The median values of each box are shown in red. The solid horizontal lines represent the clinical constraints and the dashed horizontal lines the clinical constraints with tolerance. The two largest outliers that reported low CTV coverage in the corrected ATP_1_ case are presented using red diamond markers similarly to their corresponding PTV values.

An overview of the dosimetric differences between ATS, approved ATP and corrected ATP cases for the target structures are summarized in Fig. 2.

All approved ATP cases had a V_95%_ of the CTV close to 100 %. When compared to the approved cases, corrected ATP_1_ and corrected ATP_2_ reported small underdosage to the CTV in a limited number of cases relative to the clinical constraints for a full 5 fraction treatment. A total of 93 % of the sub-fractions satisfied the planning goals for the corrected ATP_1_ and 95 % for the corrected ATP_2_ anatomies respectively. Corrected ATP_1_ reported an average of 99.7 % ± 0.5 % and corrected ATP_2_ an average of 99.7 % ± 0.4 % for the V_95%_ of the CTV. The average drop of V_95%_ of the CTV was 0.3 % for the corrected ATP_1_ and 0.2 % for the corrected ATP_2_ plans when compared to the corresponding approved ones.

For the re-created PTV contours, a drop in the coverage was also observed for the corrected ATP_1_ and corrected ATP_2_ compared to the approved plans. 89 % of the corrected ATP_1_ sub-fractions passed the clinical requirements (V_95%_ > 99 % (−3 %)), while 84 % passed the clinical requirements for the corrected ATP_2_ cases. The V_95%_ of the PTV was on average 97.9 % ± 1.6 % and 97.6 % ± 1.4 % for the corrected ATP_1_ and corrected ATP_2_ cases respectively. The V_95%_ of the PTV reported an average drop of 0.6 % for the corrected ATP_1_ and 0.7 % for the corrected ATP_2_ plans.

**Fig. 3:**
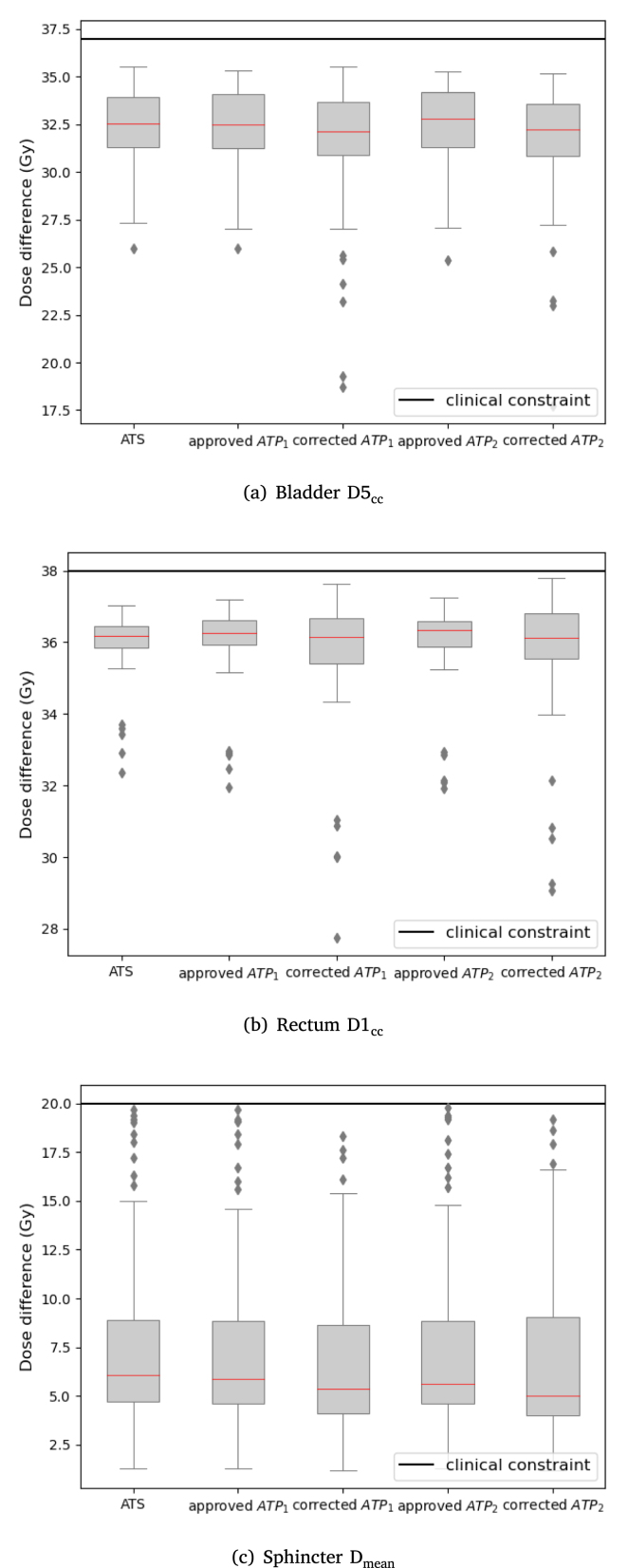
Overview of the OAR dose for the ATS, approved ATP and corrected ATP anatomies for all sub-fractions. The median values of each box are shown in red. The solid horizontal lines represent the clinical constraint values that cannot be exceeded. The range of the y-axis differs between different organ structures.

The dose distribution of the analyzed OARs was within clinically acceptable values (Fig. 3). Compared to the approved plans, the D_1cc_ of the rectum reported an average decrease of 0.4 Gy for the corrected ATP_1_ and 0.4 Gy for the corrected ATP_2_ plans. Similarly, the D_5cc_ of the bladder and the D_mean_ of the sphincter both reported an average drop of 0.7 Gy and 0.6 Gy for the corrected ATP_1_ and corrected ATP_2_ plans respectively. The dosimetric differences per sub-fraction of the approved versus corrected ATP plans for the OARs are presented in Supplementary Figure S1.

**Fig. 4:**
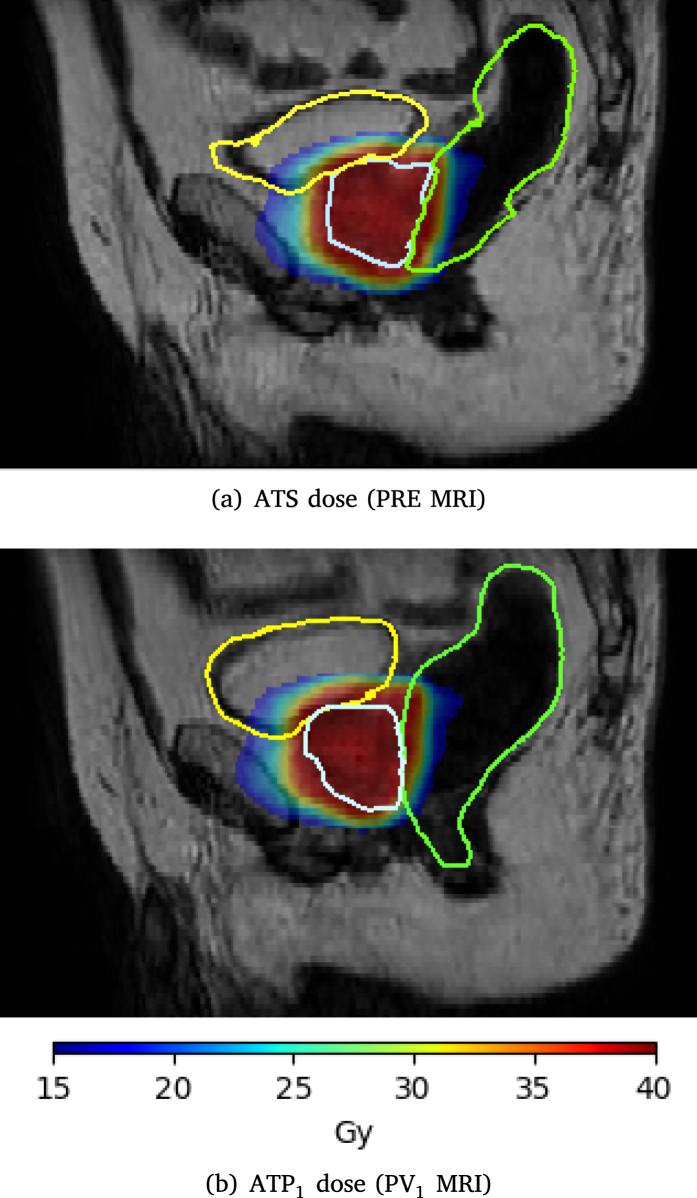
Dose distributions of the ATS (top) and corrected ATP_1_ (bottom) cases overlaid on the corresponding anatomies, PRE and PV_1_ respectively, for a sub-fraction with low CTV coverage, demonstrating the underlying anatomical deformations. CTV, rectum and bladder contours are visible on the sagittal views. The rectal deformation and large prostate rotation visible on the sagittal slice of the PV_1_ anatomy can explain the decreased CTV coverage and increased dose in the bladder.

An example of a sub-fraction with low CTV coverage is presented in Fig. 4.

## 4 Discussion

This work focused on a retrospective evaluation of clinically approved radiotherapy plans for prostate cancer patients treated with a sub-fractionation workflow. Contrary to the clinical procedure where a plan assessment was performed for each sub-fraction using rigidly adapted contours, the daily contours were deformed to match the underlying online PV anatomies. Our analysis reported high target coverage, except for a few cases that experienced intrafraction rotations and deformations that are not accounted for using the ATP steps. In addition, no overdosage was reported to any critical OAR.

Our results suggest that the majority of sub-fractions report a high CTV target coverage. The dosimetric criteria we used for our analysis at a sub-fraction level correspond to a complete treatment of 5 x 7.25 Gy fractions. For the analyzed sub-fractions only one patient received CTV dose lower than the clinical constraints in both daily sub-fractions. These observations lead us to conclude that the truly approved ATP plans for this group of patients can be characterized as clinically acceptable.

We investigated the cause of CTV coverage drop for the two largest outliers, depicted in red diamond shapes in Fig. 2. The low CTV coverage for these two cases can be mainly accredited to large intrafraction deformations. The evaluation of the OAR for these outlier cases reported higher dose in some cases, yet well within the clinical constraints. The first case reported a 2.3 Gy increase to the D_5cc_ of the bladder and a 0.2 Gy increase to the D_1cc_ of the rectum, compared to the corresponding approved dose plans. Bladder filling and rectal deformations can visually explain the dose displacement (Fig. 4). The underlying anatomical deformations and the DVH of the approved- versus corrected ATP for the second outlier case are presented in Supplementary Figures S2 and S3.

In order to further investigate a potential relationship of intrafraction drifts and dose coverage, we analyzed the target dose coverage as a function of 3D displacements, as reported from the isocenter shift in the clinical plans (Supplementary Figures S4, S5). No significant conclusions could be reached from this experiment, since the data sample was rather small, and since the cases with the largest CTV dose drops were mainly associated with large deformations and/or rotations. Additionally, the duration of the scanning time (PRE - PV_1_, PV_1_ - PV_2_) and beam-on time did not provide further indications for a drop in target coverage. Thus, we could not conclude that any of the investigated treatment plan characteristics can be used as a predictor for underdose of the target.

The main advantage of the investigated sub-fractionation workflow is the ability to deliver two sub-fractions by performing translation-based plan adaptations, thus accounting for intrafraction motion twice during a treatment session [19], [20]. Nonetheless, the ATP adaptation scheme does not account for anatomical deformations or rotations. A preliminary analysis suggests that large intrafraction rotations can occur and might lead to treatment uncertainties [16]. In order to calculate the correlation of intrafraction rotations or deformations with decreasing target coverage, more data are needed to extract useful statistics.

In our analysis we re-created the PTV structures by adding the clinically applied margins [19] to the corrected CTV contours instead of propagating the PTV contour of the PRE scan to the PV anatomies. This choice aimed at creating a meaningful PTV help structure that can reflect a safe margin around the corrected CTV to account for treatment uncertainties due to remaining intrafraction motion, including rotation. Nonetheless, the definition of PTV using traditional margin recipes is designed to ensure adequate target coverage throughout the full treatment scheme of 5 fractions and is therefore overly strict for evaluation at a sub-fraction level [23]. Hence, our reported results of PTV coverage have to be interpreted with caution, since they only reflect the plan quality of individual clinical plans and cannot serve as a direct evaluation of the clinical margins.

Our analysis has certain limitations that should be addressed in the future. First, each sub-fraction was treated as an individual plan thus far, however this is not true, as treatment margins and clinical outcomes are decided upon for the entire fractionation scheme. Therefore, interfraction dose accumulation needs to be performed for a fair assessment of the treatment margins, yet it might introduce further uncertainty due to image registration inaccuracies. Additionally, in order to assess robustness to intrafraction motion, a future evaluation could introduce the use of the INTRA MRI scan for assessment of the PV2 plan quality during the first minutes of beam-on time.

Generally ATP adaptations might be preferred over ATS steps in an online setup due to speed benefits [24], but they can potentially come at the cost of sub-optimal plan quality, as shown here. In addition, previous studies have shown that daily online re-planning has advantages over translation-only corrections and can have a major role for cases experiencing large anatomical intrafraction changes [25], [26]. Nonetheless, given its stochastic nature, prostate intrafraction motion is hard to predict [14]. A potential future solution could be the inclusion of an additional ATS step in the clinical workflow for cases where large intrafraction rotations or deformations occur, to eliminate the increased risk of sub-optimal target irradiation or late toxicity. Currently in our clinic, patients that show large motion in their first fractions are treated using larger margins in subsequent fractions, aiming at mitigating the effects of intrafraction motion.

Furthermore, adapt-to-rotations workflows have been investigated in literature as a faster alternative to ATS with comparable target coverage in the cases where the seminal vesicles are not included in the CTV [24]. The inclusion of rotational information can further offer a better way of capturing the effects of intrafraction motion. Finally, cine MRI-based motion monitoring and correction solutions are clinically available on a 1.5 T MRI radiotherapy system and can offer a faster alternative to intrafraction motion compensation via drift corrections, only when needed [27], [28].

In conclusion, this study focused on the dose-volume parameter assessment of a clinical sub-fractionation workflow using contour correction and plan re-calculations to estimate the truly approved dose distributions. Our results on CTV coverage suggest that the clinically approved ATP plans resulted in satisfying target coverage for the big majority of the plans. A future dose accumulation study can lead to more conclusions on the PTV margins used in this treatment workflow.

## CRediT authorship contribution statement

**Georgios Tsekas:** Conceptualization, Methodology, Software, Formal analysis, Writing – original draft. **Cornel Zachiu:** Conceptualization, Methodology, Software, Writing – review & editing. **Gijsbert H. Bol:** Conceptualization, Methodology, Writing – review & editing. **Jochem R.N. van der Voort van Zyp:** Writing – review & editing. **Sandrine M.G. van de Pol:** Writing – review & editing. **Johannes C.J. de Boer:** Conceptualization, Methodology, Writing – review & editing. **Bas W. Raaymakers:** Conceptualization, Methodology, Writing – review & editing.

## Declaration of competing interest

The authors declare that they have no known competing financial interests or personal relationships that could have appeared to influence the work reported in this paper.

## Appendix A Supplementary data

MMC S1 Supplementary Material.
