# Supplementary material for: Dose-volume parameter evaluation of a sub-fractionation workflow for adaptive radiotherapy of prostate cancer patients on a 1.5 T magnetic resonance imaging radiotherapy system
Source: Phys Imaging Radiat Oncol. 2025 Jan 30;33:100706. doi: 10.1016/j.phro.2025.100706 (PMC11849637; doi:10.1016/j.phro.2025.100706)
Supplement: MMC S1 — Supplementary Material. [file mmc1.pdf]

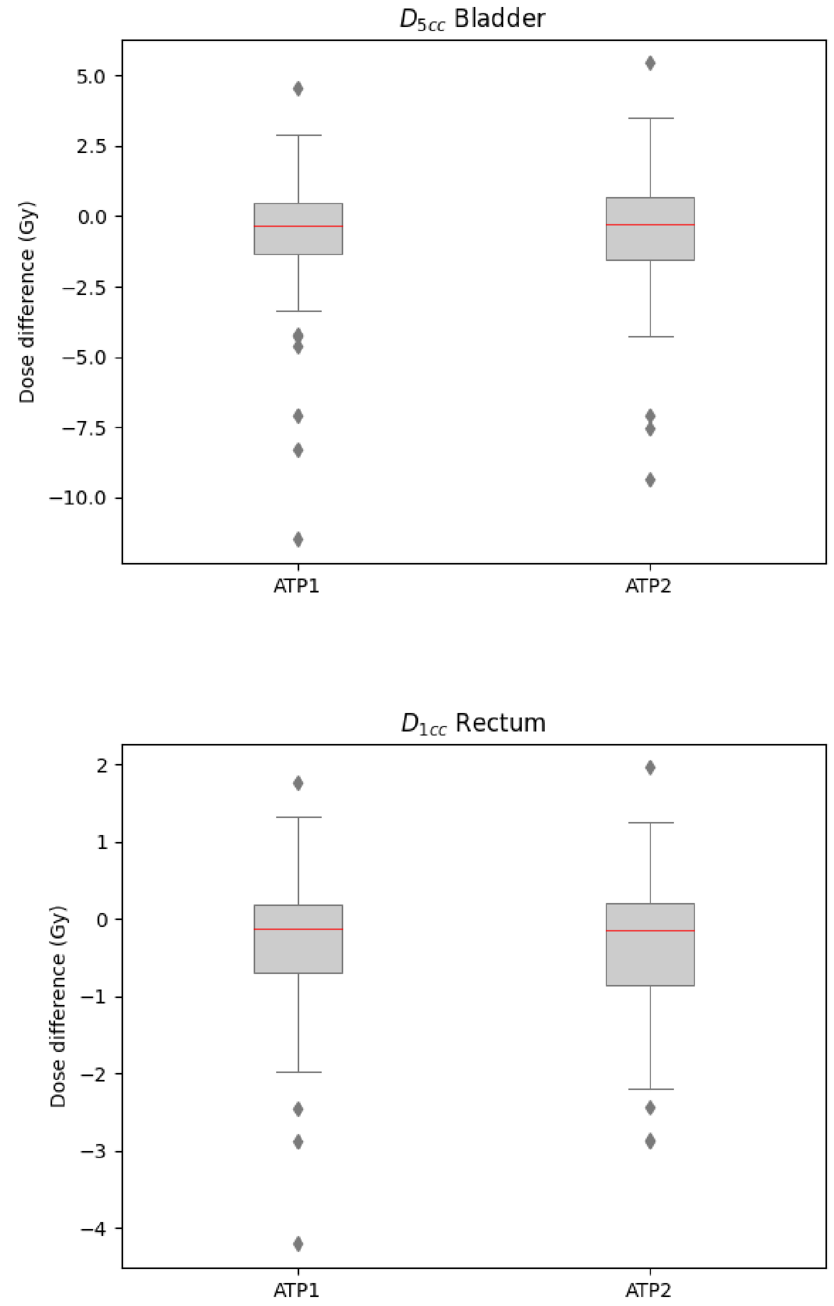

Figure S1: Dose differences for the ATP cases between approval and corrected contours for the D_{1cc} of the rectum and D_{5cc} of the bladder. The median values of each box are shown in red. A positive sign indicates a higher dose in the propagated contours compared to the corresponding approved ones. The range of the y-axis differs between different organ structures.

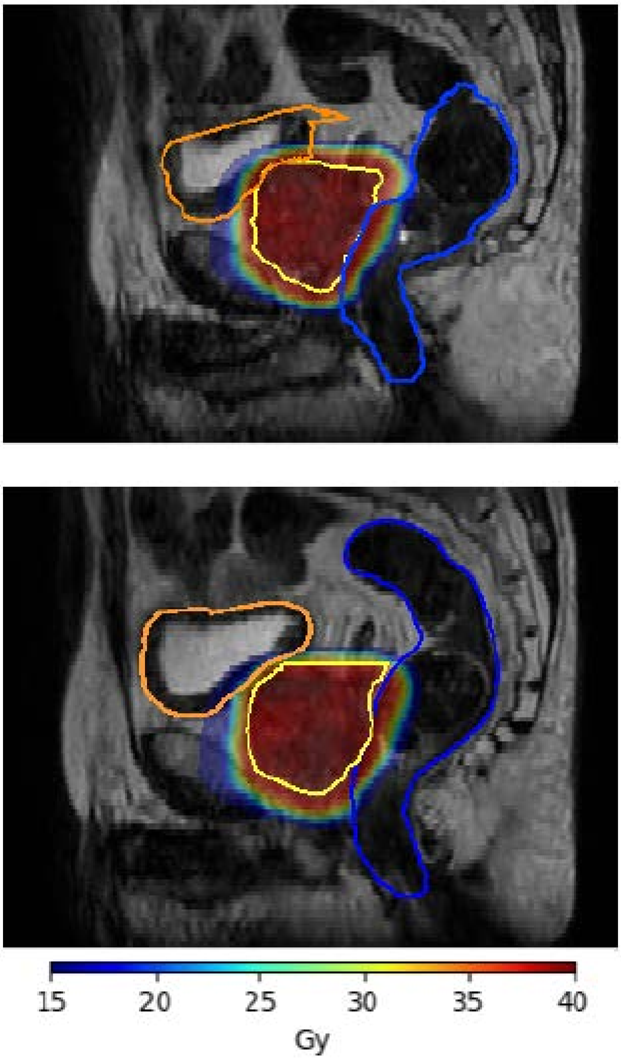

Figure S2: Dose distributions of the ATS (top) and corrected ATP_1 (bottom) cases overlaid on the corresponding anatomies, PRE and PV_1 respectively, for a sub-fraction with low CTV coverage, demonstrating the underlying anatomical deformations. CTV, rectum and bladder contours are visible on the sagittal views. Rectal deformations and bladder filling can explain the decreased CTV coverage.

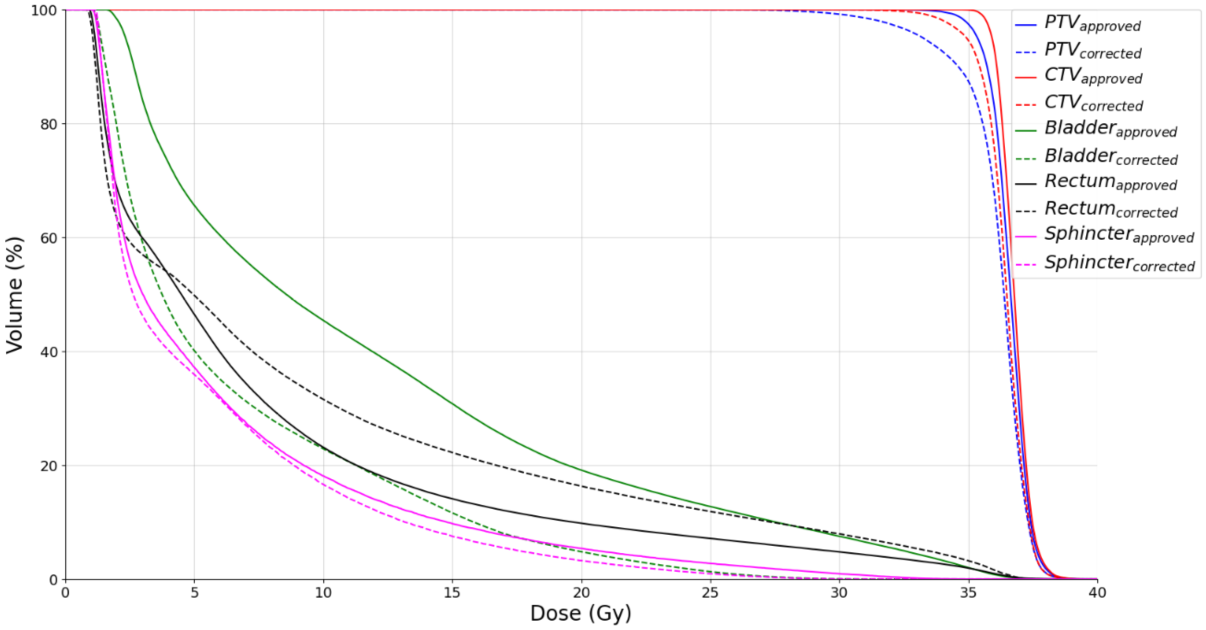

Figure S3: DVH of the approved- versus corrected ATP_1 dose distributions for the outlier case presented in Figure S2. Solid lines correspond to the approved and dashed ones to the corrected dose plans. A large dose decrease to the bladder and slight increase to the dose of the rectum can be observed when comparing the corrected case to the approved one.

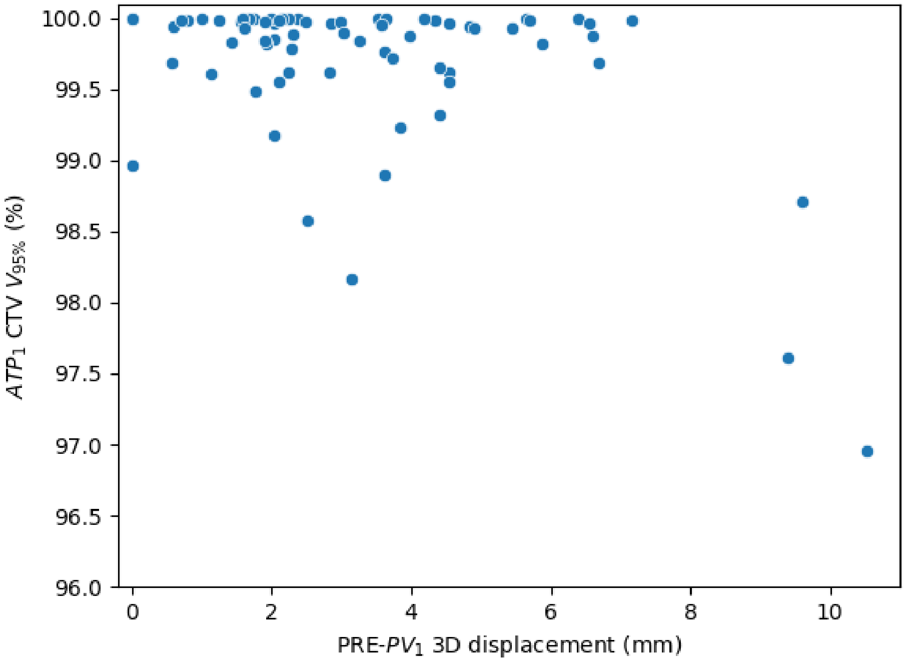

Figure S4: Infra-fraction 3D displacements between PRE and PV_1 MRI scans presented alongside with the corresponding CTV $V_{95\%}$

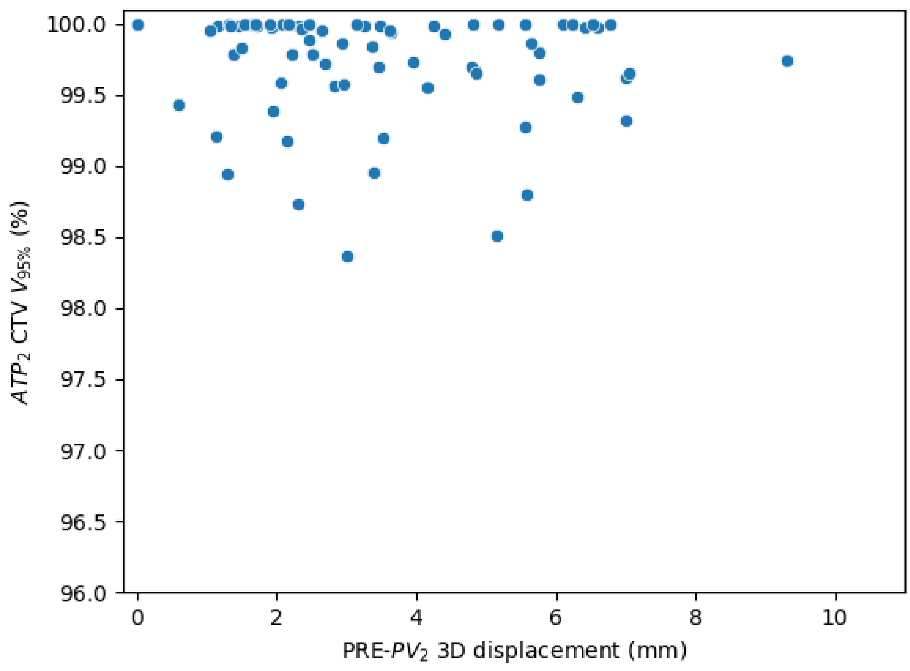

Figure S5: Infra-fraction 3D displacements between PRE and PV_2 MRI scans presented alongside with the corresponding CTV $V_{95\%}$
